# NeuroCNN_GNB: an ensemble model to predict neuropeptides based on a convolution neural network and Gaussian naive Bayes

**DOI:** 10.3389/fgene.2023.1226905

**Published:** 2023-07-27

**Authors:** Di Liu, Zhengkui Lin, Cangzhi Jia

**Affiliations:** ^1^ Information Science and Technology College, Dalian Maritime University, Dalian, China; ^2^ School of Science, Dalian Maritime University, Dalian, China

**Keywords:** neuropeptides, word2vec, one-hot, stacking strategy, convolution neural network

## Abstract

Neuropeptides contain more chemical information than other classical neurotransmitters and have multiple receptor recognition sites. These characteristics allow neuropeptides to have a correspondingly higher selectivity for nerve receptors and fewer side effects. Traditional experimental methods, such as mass spectrometry and liquid chromatography technology, still need the support of a complete neuropeptide precursor database and the basic characteristics of neuropeptides. Incomplete neuropeptide precursor and information databases will lead to false-positives or reduce the sensitivity of recognition. In recent years, studies have proven that machine learning methods can rapidly and effectively predict neuropeptides. In this work, we have made a systematic attempt to create an ensemble tool based on four convolution neural network models. These baseline models were separately trained on one-hot encoding, AAIndex, G-gap dipeptide encoding and word2vec and integrated using Gaussian Naive Bayes (NB) to construct our predictor designated NeuroCNN_GNB. Both 5-fold cross-validation tests using benchmark datasets and independent tests showed that NeuroCNN_GNB outperformed other state-of-the-art methods. Furthermore, this novel framework provides essential interpretations that aid the understanding of model success by leveraging the powerful Shapley Additive exPlanation (SHAP) algorithm, thereby highlighting the most important features relevant for predicting neuropeptides.

## Introduction

Neuropeptides are bioactive peptides that mainly exist in neurons and play a role in information transmission (Svensson et al., 2003). They are ubiquitous not only in the nervous system but also in various systems of the body, with a low content, high activity, and extensive and complex functions (Hökfelt et al., 2000). According to the specific type, they play role as transmitters, modulators, and hormones. Neuropeptides share the common characteristic that they are produced from a longer neuropeptide precursor (NPP) (Kang et al., 2019). Generally, an NPP contains a signal peptide sequence, one or more neuropeptide sequences and some other sequences that are often homologous among neuropeptides. After the NPP enters the rough endoplasmic reticulum (Rer), the signal peptide is quickly cleaved by signal peptidase and converted into a prohormone, which is transferred to the Golgi complex for proteolysis and posttranslational processing, which ultimately results in a mature neuropeptide. The neuropeptides modified by various physiological processes are transported to the terminal, stored in larger vesicles and released, and their length ranges from 3 to 100 amino acid residues (Salio et al., 2006; Wang et al., 2015). At present, there is much evidence indicating that neuropeptides play a particularly important role in the regulation of nervous system adaptation to pressure, pain, injury and other stimuli. These characteristics indicate that neuropeptides may represent a new direction in the treatment of nervous system diseases. A popular experimental method for the identification of neuropeptides is LC‒MS, whose accuracy has been greatly reduced because it has certain requirements for the quantity and quality of peptides to be extracted (Van Eeckhaut et al., 2011; Van Wanseele et al., 2016).

With the development of high-throughput next-generation sequencing technology and expressed sequence tag databases, machine learning methods have been applied to rapidly and effectively predict neuropeptide peptides. NeuroPID, NeuroPred and NeuroPP are the earliest computational tools for identifying neuropeptide precursors (Southey et al., 2006; Ofer and Linial, 2014; Kang et al., 2019). NeuroPIpred was the first predictor designed for identifying insect neuropeptides based on amino acid composition, dipeptide composition, split composition, binary profile feature extraction and the support vector machine (SVM) classification algorithm (Agrawal et al., 2019). PredNeuroP was designed by building a two-layer stacking model that was trained on nine kinds of hybrid features for animal phyla neuropeptide prediction (Bin et al., 2020). In PredNeuroP, extremely randomized trees (ERT), artificial neural network (ANN), k-nearest neighbor (KNN), logistic regression (LR), and extreme gradient boosting (XGBoost) were employed to develop ML-based models. In terms of feature coding, PredNeuroP uses amino acid composition, dipeptide composition, binary profile-based features, amino acid index features, grouped amino acid composition, grouped dipeptide composition, composition-transition-distribution, and amino acid entropy. In 2021, Hasan *et al.* developed a meta-predictor NeuroPred-FRL on the basis of 11 different encodings and six different classifiers (Hasan et al., 2021). Although the existing models have achieved relatively satisfactory prediction performances, most of them are developed based on traditional machine learning methods, and deep learning predictors have not been fully explored.

In this work, we have made a systematic attempt to create a tool that can predict neuropeptides using a stacking strategy based on four convolution neural network models. These base models were separately trained on one-hot encoding, AAIndex, G-gap dipeptide encoding and word2vec. By comparing five integration strategies, including LR (Perlman et al., 2011), AdaBoost (Freund and Schapire, 1997), GBDT (Lei and Fang, 2019), Gaussian NB and XGBoost, on 5-fold cross-validation tests, we finally selected Gaussian NB to construct our predictor designated NeuroCNN_GNB, with an AUC of 0.963, Acc of 90.77%, Sn of 89.86% and Sp of 91.69% on 5-fold cross-validation test. Moreover, to enhance the interpretability of the ‘black-box’ machine learning approach used by NeuroCNN_GNB, we employed the Shapley Additive exPlanation (SHAP) method (Lundberg and Lee, 2017) to highlight the most important and contributing features allowing NeuroCNN_GNB to generate the prediction outcomes. The analysis results showed that one-hot encoding and word2vec play key roles in the identification of neuropeptides.

## Materials and methods

### Overall framework

**FIGURE 1:**
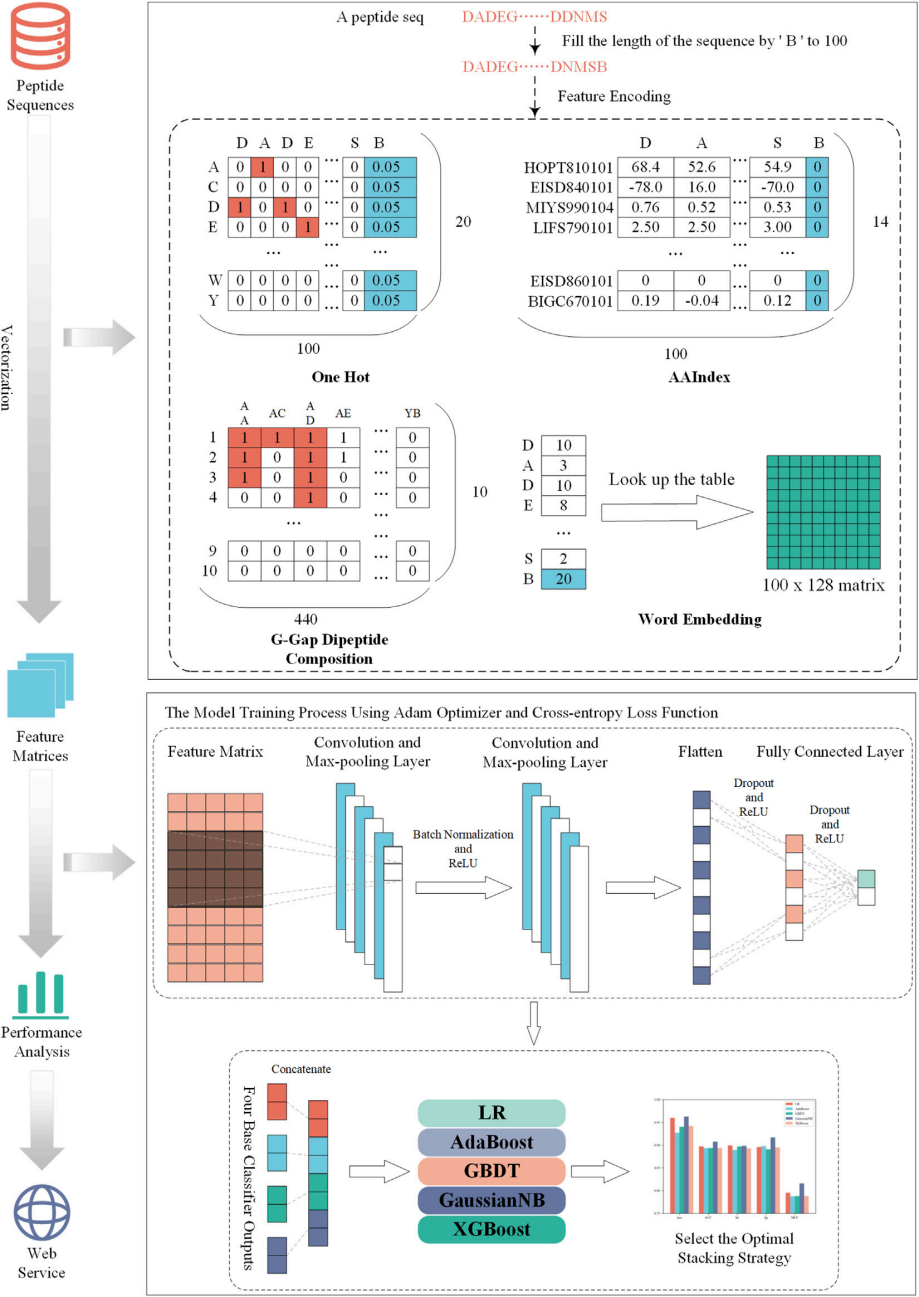
The developmental flowchart of NeuroCNN_GNB.

The construction process of NeuroCNN_GNB is shown in Figure 1. First, we collected the training dataset and the independent test dataset from original work (Bin et al., 2020). Then, we extracted four types of sequence information from different aspects and combined them with convolutional neural networks to construct base classifiers. In the third step, we considered different stacking strategies to build the final optimal model. Next, we evaluated the performance of the model on the training and independent test datasets and compared it with that of other state-of-the art methods. In the final step, the NeuroCNN_GNB webserver and the corresponding source code were developed and publicly released.

### Data collection

Building the benchmark datasets is one of the most important and critical steps in building a prediction algorithm. In this work, we applied the dataset that was first constructed by (Bin et al., 2020) and subsequently used by (Hasan et al., 2021; Jiang et al., 2021). This dataset contains 2425 neuropeptides collected from (Wang et al., 2015) and 2425 nonneuropeptides collected from Swiss-Prot (UniProt Consortium, 2021). It should be noted that the samples in this dataset were processed in two steps. The first step was to remove those protein sequences that contained less than 5 and more than 100 amino acids, as neuropeptides are small peptides generally containing less than 100 amino acids (Salio et al., 2006; Wang et al., 2015). The second step was to remove the protein sequences with a high similarity. Using the threshold of 0.9, CD-HIT was applied to delete redundant samples inside positive and negative samples, and CD-HIT-2D was applied to delete redundant samples between positive and negative samples (Huang et al., 2010). To optimize and compare the predictor, the dataset was further divided into training and independent test datasets according to the proportion of 8:2.

### Feature extraction

In this study, we use four different encoding schemes to obtain information on neuropeptides and nonneuropeptides, including one-hot encoding, physicochemical-based features, amino-acid frequency-based features, and embedding methods. These encoding schemes consider 20 types of natural amino acid residues (‘A', ‘C', ‘D', ‘E', ‘F', ‘G', ‘H', ‘I', ‘K', ‘L', ‘M', ‘N', ‘*p*', ‘Q', ‘R', ‘S', ‘T', ‘V', ‘W','Y') and add a pseudo character (‘B') to obtain the characteristics with the same dimension. Specifically, we fixed the sequence length to 100 and filled the gaps with ‘B' if the protein sequence length was less than 100. The details of the feature encodings are described in the following sections.

### One-hot encoding

One-hot encoding can reflect the specific amino acid position of a given protein sequence. Each amino acid residue was transformed into a binary vector as follows:

$$\begin{array}{c} \left\{\begin{array}{c} A=\left(1,0,0,\ldots ,0,0\right)\\ C=\left(0,1,0,\ldots ,0,0\right)\\ \ldots \\ \ldots \\ W=\left(0,0,0,\ldots ,1,0\right)\\ V=\left(0,0,0,\ldots ,0,1\right)\\ B=\left(0.05,0.05,0.05,\ldots ,0.05,0.05\right) \end{array}\right. \end{array}$$ (1)

The reason that we set each element of B as 0.05 is that we assumed the average frequency of each amino acid is uniformly distributed as the work (Pan et al., 2018; Pan and Shen, 2018; Yang et al., 2021). Thus, one-hot encoding generates a 100 × 20-D feature matrix for a given peptide sequence with a length of 100.

### Amino acid index (AAIndex)

AAIndex is a database that includes 566 various physicochemical and biochemical properties of amino acids and amino acid pairs (Kawashima et al., 2007). In this section, we chose 14 properties because they have been verified to be very effective in improving the prediction performance of neuropeptide recognition (Bin et al., 2020; Khatun et al., 2020). Their accession numbers are HOPT810101, EISD840101, MIYS990104, LIFS790101, MAXF760101, CEDJ970104, GRAR740102, KYTJ820101, MITS020101, DAWD720101, BIOV880101, CHAM810101, EISD860101, and BIGC670101. For each physicochemical property, each amino acid was assigned a numerical index, and their values are listed in Supplementary Table S1.

### G-gap dipeptide encoding

The G-gap dipeptide encoding scheme incorporates the amino acid frequency information of the peptide sequence, where g’ is a parameter that represents a dipeptide with a gap of ‘g’ amino acids (A, C, D, E, F, G, H, I, K, L, M, N, P, Q, R, S, T, V, W, Y, B) (Lin et al., 2013; Lin et al., 2015; Xu et al., 2018). In this study, we tried 0, 1, 2,3, and 4-gap dipeptides to encode each protein peptide. For the 21 amino acids (20 natural amino acids and a temporary amino acid B′), there were 441 dipeptide combinations. We discarded the combination BB’ and reserved 440 amino acid pairs to effectively capture the component information in protein peptides. Based on the statistical analysis, the highest number of amino acid pairs in the existing training dataset was 10. Therefore, the number of amino acid pairs was encoded into one-hot encoding of 10 dimensions. Finally, we could generate a characteristic matrix of 440*10 for a given peptide sequence.

### Word embedding

Word embedding is a strategy to convert words in text into digital vectors for analysis using standard machine learning algorithms (Mikolov et al., 2013). This strategy has been extensively applied in natural language processing and has been introduced to the fields of proteomics and genomics (Lilleberg et al., 2015; Ng, 2017; Jatnika et al., 2019; Wu et al., 2019). Word2vec is an efficient method to create word embedding that includes two algorithms, namely, skip Gram and CBOW (continuous bag-of-words). The difference between them is that skip Gram predicts the words around the head word through the central word, while CBOW predicts the central word through the surrounding words. According to the preliminary experimental performance, we selected skip Gram to encode each protein peptide in the subsequent experiments.

### Model framework

To capture the information contained in multiple feature scenarios, we used a stacking strategy to develop our model to efficiently identify neuropeptides. Stacking is an ensemble learning method that combines predicted information from multiple models to generate a more stable model (Ganaie et al., 2022). The stacking method has two main steps, in which we used the so-called base classifier and meta-classifier. In our work, four base classifiers were constructed based on convolutional neural networks (CNNs). For each type of feature, the corresponding CNN model was trained using grid search to optimize the hyperparameters. All training processes are conducted through the Python package ‘pytorch'.

### Performance evaluation

To objectively evaluate and compare the predictive performance of the models, five frequently used performance metrics were used, including sensitivity (Sn), specificity (Sp), accuracy (Acc), and MCC. Their formulas are given as follows:

$$\begin{array}{c} Sn=\frac{TP}{TP+FN} \end{array}$$ (2)

$$\begin{array}{c} Sp=\frac{TN}{TN+FP} \end{array}$$ (3)

$$\begin{array}{c} Acc=\frac{TP+TN}{TP+TN+FP+FN} \end{array}$$ (4)

$$\begin{array}{c} MCC=\frac{TP\times TN-FP\times FN}{\sqrt{\left(TP+FP\right)\times \left(TP+FN\right)\times \left(TN+FP\right)\times \left(TN+FN\right)}} \end{array}$$ (5)

where TP, TN, FP and FN denote the numbers of true positives, true negatives, false-positives and false-negatives, respectively. Furthermore, we used the area under the ROC curve (AUC) as one of the main metrics to evaluate model performance.

## Results and discussion

### Performance analysis of base classifiers

**FIGURE 2:**
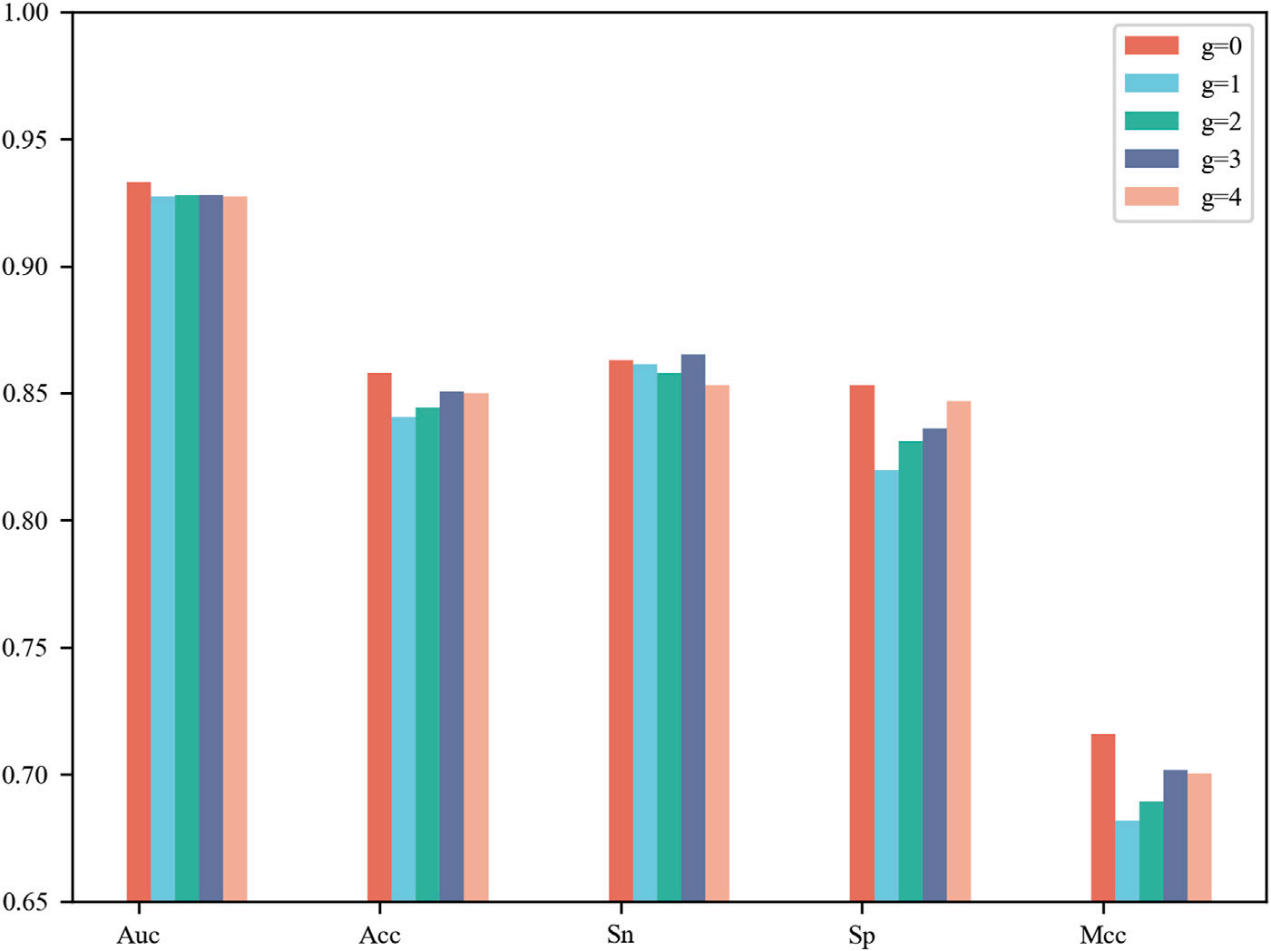
Performance comparison of g-Gap Model on 5-fold cross-validation test.

CNN contains a number of tunable hyperparameters, which can affect the validity and robustness of the model. We used a grid search to tune the hyperparameters and explore their optimal combination using 5-fold cross-validation. The average AUCs were designed as the criterion for selecting the parameter combinations. For the G-gap-model (g = 0, 1, 2, 3, 4), we compared their performance on 5-fold cross-validation and show their results in Figure 2. The model based on g = 0 reached the best AUC of 0.933, Acc of 0.858, Sp of 0.853 and MCC of 0.716, while the model based on g = 3 achieved the best Sn of 0.865. Upon comprehensive consideration, an appropriate selection of g = 0 was adopted to build one of the base classifiers. The details of the G-gap based model are summarized in Supplementary Table S3.

Supplementary Table S2 summarizes the optimal combination of parameters for each base classifier, and Table 1 lists their 5-fold cross-validation results. It was observed that the one-hot-based model achieved the best AUC of 0.956, which was slightly superior to the AAIndex and word2vec models. In total, the AUC values of the four base classifiers were greater than 0.93, showing satisfactory prediction results.

**TABLE 1:** The Performance of base classifiers on 5-fold cross validation.

Feature	AUC	Acc	Sn	Sp	MCC
One-Hot	0.956	0.887	0.891	0.883	0.775
AAIndex	0.954	0.885	0.872	0.899	0.771
G-Gap	0.933	0.858	0.863	0.853	0.716
Word2vec	0.952	0.882	0.867	0.898	0.765

In addition, we also performed 10-fold cross-validation test to evaluate the generalization ability of our model. As shown in Table 2, there is almost no difference in the prediction results between 5-fold and 10-fold cross-validation results. Specifically, the AUC of 10-fold cross-validation results based on one-hot is 0.004 lower, based on AAIndex is 0.006 lower, based on word2vec is 0.01 lower than that of 5-fold, respectively.

**TABLE 2:** Results of 5-fold and 10-fold cross-validation on base classifiers.

Cross-validation	Encoding	AUC	Acc	Sn	Sp	MCC
5-fold	one-hot	**0.956**	**0.887**	**0.891**	0.883	**0.775**
10-fold	one-hot	0.952	0.882	0.879	**0.885**	0.765
5-fold	AAIndex	**0.954**	**0.885**	**0.872**	**0.899**	**0.771**
10-fold	AAIndex	0.948	0.877	0.868	0.885	0.755
5-fold	word2vec	**0.952**	**0.882**	**0.867**	**0.898**	**0.765**
10-fold	word2vec	0.942	0.871	0.865	0.875	0.741

The bold values indicate the higher values of the 5-fold and the 10-fold cross validation results.

### Stacking models providing robust and reliable prediction results

**FIGURE 3:**
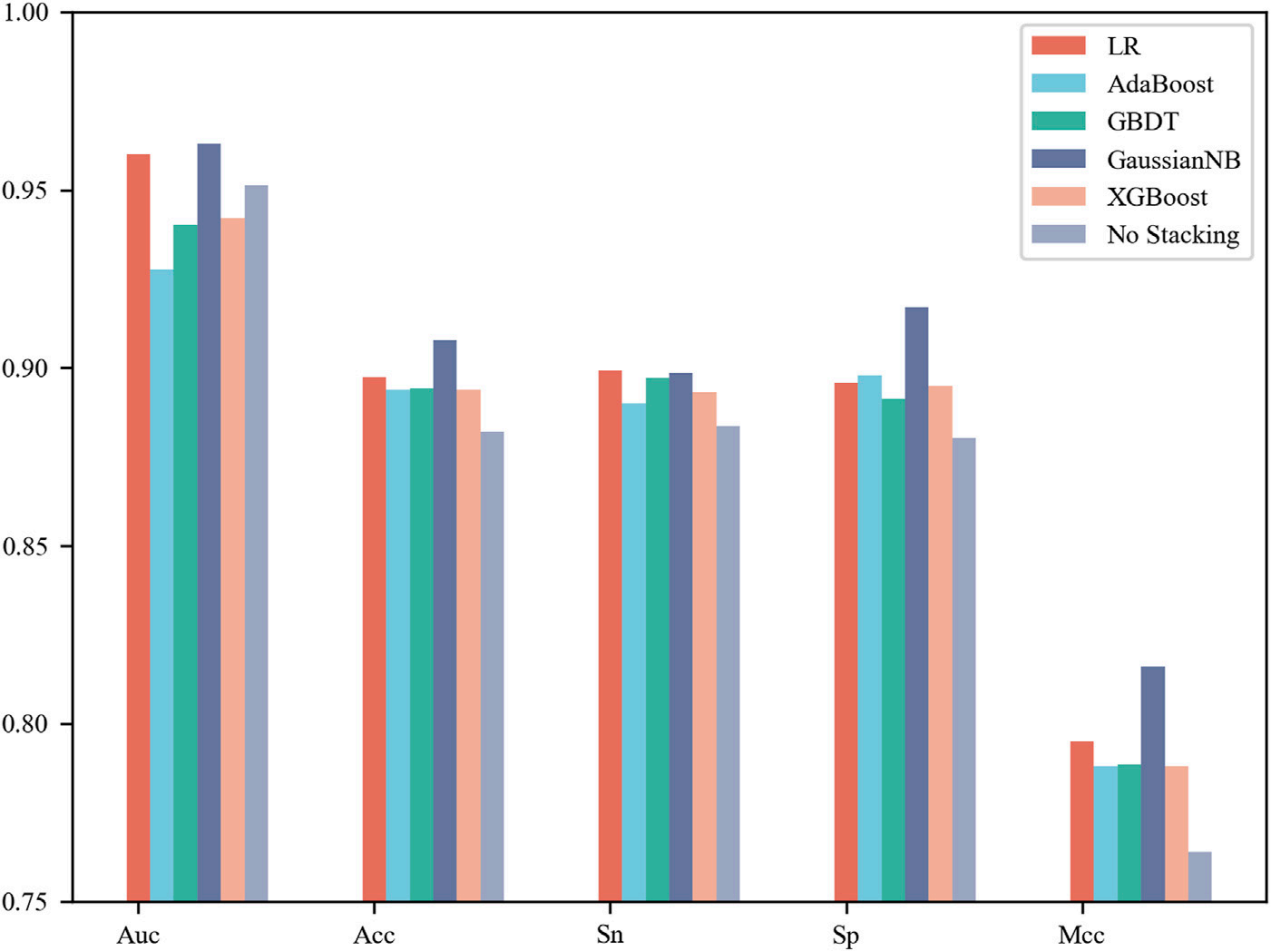
Performance comparison of the different stacking algorithms on 5-fold cross-validation test.

In this section, each base classifier was considered a weak classifier and then integrated into a strong classifier. LR, AdaBoost, GBDT, Gaussian NB and XGBoost were used as stacking algorithms to construct the meta model. The specific process is that we concatenate the prediction results of four base classifiers for the same sample as the input to the stacking algorithm to obtain the final classification label (Rokach, 2010; Lalmuanawma et al., 2020; Aishwarya and Ravi Kumar, 2021; Ganaie et al., 2022). It can be observed from Figure 3 that Gaussian NB achieved the best performance with an AUC of 0.963, Acc of 90.77%, Sn of 89.86% and Sp of 91.69% on the 5-fold cross-validation test. Moreover, this set of results achieved by the stacking strategy was better than those obtained by the four base classifiers. However, not all integration results were superior to a single model. The stacking results of AdaBoost were inferior to those of the four base classifiers, whose AUC was only 0.928. Taken together, the results showed that selection of a stacking strategy is necessary for different biological sequences. How to find the relationship between the data distribution and classification algorithm is a problem worth studying in the future.

### Performance comparison with existing methods on the independent test datasets

We then used the independent test dataset to verify the robustness of NeuroCNN_GNB and compared the prediction results with those of NeuroPpred-Fuse, NeuroPred-FRL and PredNeuroP. These predictors were developed based on the same training dataset as our model, which guarantees the fairness and objectivity of the independent test. The comparison results in Table 3 show that our model obtained the best AUC of 0.962, Acc of 0.918 and MCC of 0.836, which implied a similar effect of predicting positive and negative samples. NeuroPred-FRL achieved the second best AUC of 0.960 and the best Sn of 0.929, and NeuroPred-Fuse showed the best Sp of 0.930. Thus, each of the three models has its own advantages in prediction performance based on four types of features and four base classifiers, whose complexity was lower than that of the other four models. In particular, this work not only establishes an efficient prediction model but also provides a freely convenient web server for researchers.

**TABLE 3:** Comparing with other exiting methods on the independent test dataset.

Method	AUC	Acc	Sn	Sp	MCC
NeuroPred-FRL	0.960	0.916	**0.929**	0.903	0.834
NeuroPpred-Fuse	0.958	0.906	0.882	**0.930**	0.813
PredNeuroP	0.954	0.897	0.886	0.907	0.794
Our model	**0.962**	**0.918**	0.919	0.917	**0.836**

### Visualization of features

**FIGURE 4:**
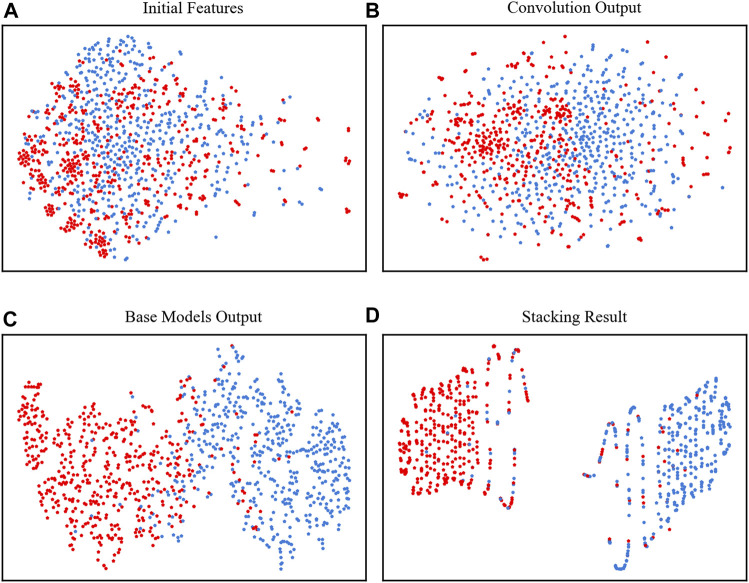
t-SNE plots of the positive and negative samples. **(A)** The initial features, **(B)** the features extracted by convolutional layer, **(C)** outputs of the four base classifiers and **(D)** the final output of the model.

To clearly show how the model performs at each stage, we used t-SNE to visually observe the classification results of the two types of data (Van der Maaten and Hinton, 2008). In Figure 4A, the points were mixed in disorder by using the initial features to concatenate all 4 kinds of encodings, which were almost impossible to divide. However, after the four base classifiers, the neuropeptides and nonneuropeptides were almost separated except for the middle part, which occasionally overlaps, as shown in Figures 4B, C. Finally, after the stacking strategy, our model clearly identified the neuropeptides and nonneuropeptides, as shown in Figure 4D. This figure shows that our model can effectively acquire the intrinsic information of the neuropeptides.

### Model interpretation: the effect of feature encoding on model prediction

In this study, four different feature-encoding schemes were used to generate the feature vectors. The performance of each type of feature is listed in Table 1. To display the influence of various features on the model more intuitively, the SHAP (SHapley Additive exPplanation) algorithm was applied to evaluate feature behavior in our datasets (Lundberg and Lee, 2017).

**FIGURE 5:**
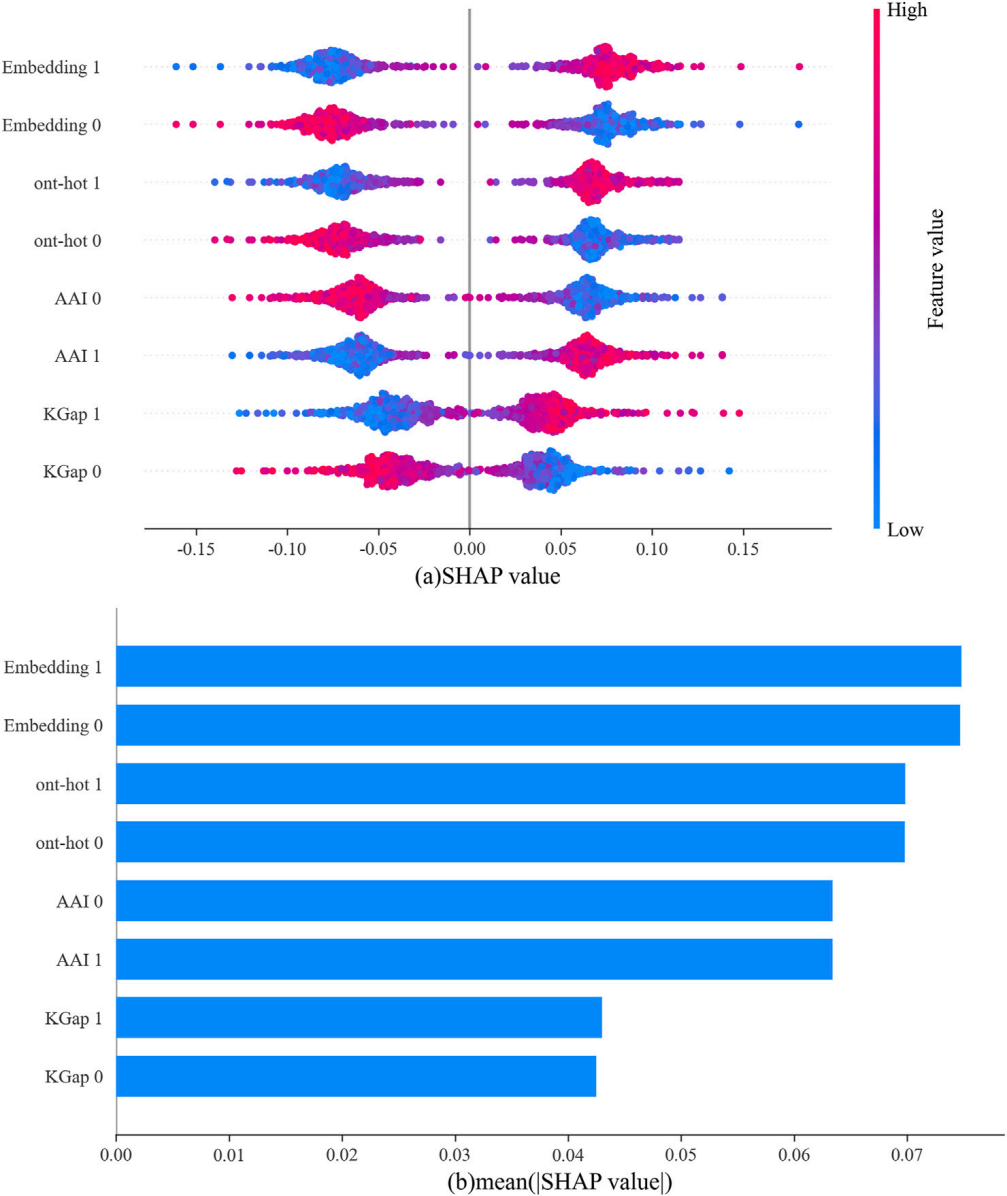
The outputs from four base classifiers according to SHAP values for the neuropeptides, **(A)** SHAP value for each sample; **(B)** the average of the absolute values of SHAP for all samples.

In Figure 5A, the abscissa represents the SHAP value, the ordinate represents each type of feature for the positive sample (abbreviated as 1) and negative sample (abbreviated as 0), and each point is the SHAP value of an instance. Redder sample points indicate that the value of the feature is larger, and bluer sample points indicate that the value of the feature is smaller. If the SHAP value is positive, this indicates that the feature drives the predictions toward neuropeptides and has a positive effect; if negative, the feature drives the predictions toward nonneuropeptides and has a negative effect. For a more intuitive display, the average absolute values for each type of feature are shown in Figure 5B. It can be clearly observed that among the output of the four base classifiers, the one-hot and word embedding-based models were the primary contributors to the final output of the model.

## Conclusion

In this study, we introduced a robust predictor based on a stacking strategy to accurately predict neuropeptides. The predictor extracted four types of protein sequence information, employed CNN to train base classifiers, and then selected Gaussian NB to build an ensemble model. The validity of our model was assessed using 5-fold cross-validation and an independent test dataset. In addition, t-SNE was used to visually observe the clustering of samples at each stage, and SHAP was also used to interpret what role each type of feature plays in the classification process. A user-friendly webserver and the source code for our model are freely available at http://47.92.65.100/neuropeptide/. Our model showed satisfactory results when evaluated from different aspects, but there is still room for optimization of the model as a predictor with the increase in experimental neuropeptide data.

## Data Availability

The original contributions presented in the study are included in the article/Supplementary Material, further inquiries can be directed to the corresponding author.
