# Supplementary material for: NeuroCNN_GNB: an ensemble model to predict neuropeptides based on a convolution neural network and Gaussian naive Bayes
Source: Front Genet. 2023 Jul 27;14:1226905. doi: 10.3389/fgene.2023.1226905 (PMC10414792; doi:10.3389/fgene.2023.1226905)
Supplement: Supplementary file 1 [file Table2.DOCX]

Table S1. The 14 properties of amino acids.

| **Description** | **A** | **R** | **N** | **……** | **W** | **Y** | **V** |
| --- | --- | --- | --- | --- | --- | --- | --- |
| BIGC670101 | 52.6 | 109.1 | 75.7 | …… | 135.4 | 116.2 | 85.1 |
| BIOV880101 | 16 | -70 | -74 | …… | 145 | 53 | 123 |
| CHAM810101 | 0.52 | 0.68 | 0.76 | …… | 0.7 | 0.7 | 0.76 |
| DAWD720101 | 2.5 | 7.5 | 5 | …… | 7 | 7 | 5 |
| EISD840101 | 0.25 | -1.76 | -0.64 | …… | 0.37 | 0.02 | 0.54 |
| EISD860101 | 0.67 | -2.1 | -0.6 | …… | 2.6 | 1.6 | 1.5 |
| GRAR740102 | 8.1 | 10.5 | 11.6 | …… | 5.4 | 6.2 | 5.9 |
| HOPT810101 | -0.5 | 3 | 0.2 | …… | -3.4 | -2.3 | -1.5 |
| KYTJ820101 | 1.8 | -4.5 | -3.5 | …… | -0.9 | -1.3 | 4.2 |
| LIFS790101 | 0.92 | 0.93 | 0.6 | …… | 1.54 | 1.53 | 1.81 |
| MAXF760101 | 1.43 | 1.18 | 0.64 | …… | 1.01 | 0.69 | 0.98 |
| CEDJ970104 | 7.9 | 4.9 | 4 | …… | 1.2 | 3.1 | 6.8 |
| MITS020101 | 0 | 2.45 | 0 | …… | 6.93 | 5.06 | 0 |
| MIYS990104 | -0.04 | 0.07 | 0.13 | …… | -0.33 | -0.29 | -0.29 |

Table S2. The best optimal hyper-parameter combination for each base classifier.

| **Feature-based** | **Feature dimension** | **Learning Rate** | **Weight Decay** | **T_max** | **AUC** |
| --- | --- | --- | --- | --- | --- |
| One-hot | 100*20 | 0.0005 | 0.06 | 25 | 0.956 |
| AAIndex | 100*14 | 0.0005 | 0.02 | 25 | 0.954 |
| g-Gap | 440*10 | 0.0006 | 0.04 | 35 | 0.933 |
| Word2vec | 100*128 | 0.0004 | 0.02 | 25 | 0.952 |

Table S3. The results of G-Gap of base classifier on 5-fold cross-validation.

| **G-Gap** | **AUC** | **Acc** | **Sn** | **Sp** | **MCC** |
| --- | --- | --- | --- | --- | --- |
| 0 | 0.933 | 0.858 | 0.8628 | 0.8531 | 0.716 |
| 1 | 0.9272 | 0.8405 | 0.8613 | 0.8196 | 0.6818 |
| 2 | 0.928 | 0.8443 | 0.8578 | 0.8312 | 0.6894 |
| 3 | 0.928 | 0.8505 | 0.8652 | 0.8362 | 0.7016 |
| 4 | 0.9274 | 0.85 | 0.853 | 0.8468 | 0.7004 |
| 5 | 0.9095 | 0.8289 | 0.8527 | 0.8052 | 0.6587 |
| 6 | 0.9158 | 0.8373 | 0.8484 | 0.8262 | 0.675 |
| 7 | 0.9100 | 0.8289 | 0.8510 | 0.8065 | 0.6583 |
| 8 | 0.9077 | 0.8320 | 0.8514 | 0.8122 | 0.6645 |
| 9 | 0.9071 | 0.8263 | 0.8444 | 0.8081 | 0.6530 |
